# Performance and functional assessment of the Kimera P-IV point-of-care plasmonic qPCR prototype for ultra rapid pathogen detection of *chlamydia trachomatis*

**DOI:** 10.1017/S0950268825000081

**Published:** 2025-01-30

**Authors:** Joshua Hayes, Seung Soo Lee, Jason Carnevale, Daniel Shamir, Marc Bohbot, Andrew G. Kirk, Miltiadis Paliouras, Mark A. Trifiro

**Affiliations:** 1 Lady Davis Institute for Medical for Medical Research – Jewish General Hospital, Montreal, QC, Canada; 2 Division of Experimental Medicine, McGill University, Montreal, QC, Canada; 3 Department of Biology, Concordia University, Montreal, QC, Canada; 4 Nexless Healthcare LP, Montreal, QC, Canada; 5 Department of Electrical and Computer Engineering, McGill University, Montreal, QC, Canada; 6 Department of Medicine, McGill University, Montreal, QC, Canada

## Abstract

Current standard microbiological techniques are generally very time consuming, usually requiring 24–72 h to establish a diagnosis. Consequentially, contemporary clinical practices implement broad-spectrum antibiotic administration prior to pathogen detection, prompting the emergence of extremely dangerous antibiotic-resistant bacteria. Additionally, lengthy test-to-result turnover times can greatly exacerbate the rate of disease spread. Rapid point-of-care (POC) diagnostics has quickly gained importance since the SARS-CoV-2 pandemic; accordingly, we have developed a rapid four-channel POC plasmonic quantitative polymerase chain reaction (qPCR) machine (Kimera P-IV) to respond to the deficiencies in infection control. Utilizing gold nanorods (GNRs) as nano-heaters and integrating vertical cavity surface emitting lasers (VCSEL) to replace traditional Peltier blocks, the Kimera P-IV has also incorporated quantitative real-time fluorescent monitoring. Using *Chlamydia trachomatis* genetic material to evaluate the rapid thermocycling performance of the platform, we have generated positive amplicons in less than 13 min; however, to achieve these results, several biological reagent considerations needed to be taken into account, specifically primer design. The device can achieve a limit of detection (LoD) of <10^1^ DNA copies, a PCR efficiency of 88.3%, and can differentiate positive from negative results with 100% accuracy. Moreover, it can also analyze *C. trachomatis* DNA spiked urine samples via a simple dilution, suggesting that a separate nucleic acid step may not be needed for diagnosing infections. In conclusion, the operation of the Kimera P-IV prototype places it in a unique position of POC devices to revolutionize infectious disease diagnosis.

## Introduction

Infectious diseases remain the single most important health-related issue worldwide despite enormous strides made in public health initiatives, antimicrobial drugs, and vaccine development. Infectious microbiological agents overwhelmingly account for most of the mortality and morbidity in healthy younger individuals and usually account for mortality in older individuals with significant unrelated diseases [1]. In 2010, 15 million deaths were attributed to infectious agents, and the World Health Organization forecasts infectious-related deaths to be above 20 million annually by the year 2050 [1].

Clinical microbiology is performed in a central laboratory and is an increasingly costly and slow process, often requiring 48–72 h from test to result. Rare organisms can take much longer, as some microorganisms may require very unusual culture conditions. Recently, a large multicenter study highlighted the importance of timely implementation of antibiotics. In critically ill patients, every hour of delay was associated with a 1.09 relative risk increase in mortality [2]. Therefore, many of the shortcomings of classical microbiology can be addressed by employing pathogen detection by genotyping the organism. Almost all human pathogens have had their DNA genome sequenced; microbial DNA sequence has even superseded classical microbiology to define phylogeny. Thus, PCR can readily fulfill the role of genetic recognition of human pathogens [3, 4]. A PCR approach to pathogen detection was first used to identify tuberculosis, a scarce organism that was extremely difficult to isolate and grow [5]. PCR can also be used to verify antibiotic resistance which is mediated by bacterial plasmids with specific DNA sequences [6–9].

Within the last few years, PCR has been increasingly established as a valuable tool for the clinical diagnosis of both bacterial and viral infections, with SARS-CoV-2 being a prime example [3, 10, 11]. Result outcomes are much faster than classical microbiology, but still fall into the same trappings of employing centralized laboratories, with many test-to-results times reaching 24 h (although the fastest times can get a few hours depending on the PCR test). If acute infectious disease diagnostics can utilize a point-of-care (POC) platform, physicians can provide more effective care. POC testing would greatly assist antibiotic stewardship and infection control. The COVID-19 pandemic and access to and demand for testing have decreased since 2020, further highlighting the need for accessible testing [12]. Sexually transmitted disease (STD) clinics would greatly benefit from POC rapid and sensitive testing as *Chlamydia trachomatis* (CT) is one of the sexually transmitted pathogens for which PCR testing has been approved by the FDA, and through rapid testing, the blind administration of broad-spectrum antibiotics can be mitigated [13].

The POC plasmonic qPCR machine, Kimera P-IV, aims to tackle these issues. When all channels are running, the device can complete 30 cycles of PCR in under 13 min. The Kimera P-IV has two unique components that are key to its superior efficiency, which is the integration of gold nanorods (GNRs) and vertical-cavity surface-emitting lasers (VCSEL). In contrast to Peltier heating blocks that are used to manage the temperature in a conventional thermocycler, the plasmonic PCR technology utilizes GNRs within the samples as nano heaters to manage the temperatures at desired levels. In a typical reaction, the GNRs are excited by a VCSEL and convert light to thermal energy with approximately 99% efficiency [14], allowing very rapid heating times. Using *C. trachomatis* as the test infectious pathogen, we highlight the performance of the Kimera P-IV real-time quantitative DNA amplification features that will provide valuable clinical and research information, as well as a near-immediate test-to-result turnover time.

## Materials and Methods

### Reaction mixture and thermocycling conditions

The reaction mixture was designed specifically for the plasmonic PCR. Each 20 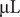 sample comprises the components listed in Table 1. PEGylated gold nanorods (GNR) were purchased from Nanopartz (Loveland, CO, USA) and used at a concentration of 2.5 nM per reaction, as outlined by Mohammadyousef *et al*. [15]. Other reagents included Hemo Klen Taq (New England Biolabs Canada, Whitby, ON, Canada), SYTO-16, dNTPs (Thermo Fisher Scientific, Saint-Laurent, QC, Canada), and different sets of primers (Integrated DNA Technologies IDT, Coralville, IA, USA). Quantitative *C. trachomatis* Strain LGV III DNA was purchased from the American Type Culture Collection (Manassas, VA, USA), and supplied with a CoA containing information regarding genomic copy number. The list of primers designed for the *C. trachomatis* cryptic plasmid (CTC) is listed in Table 2. In the context of plasmonic PCR, proper primer design has greatly mitigated the primer dimerization phenomenon, thereby increasing the sensitivity and specificity of the diagnosis. All primers were designed and assessed on IDT’s OligoAnalyzer Tool.

**Table 1.** PCR reagent mixes

Reagent	Volume (μL)	Final concentration
5× reaction buffer	4	1×
DNA polymerase	1	Unknown^a^
GNRs	1	2.5 nM^b^
SYTO–16	0.08	4 mM
Purified DNA	1	Diluted sample^c^
Forward primer	0.6	0.3 μM
Reverse primer	0.6	0.3 μM
dNTPs	0.5	250 μM
Milli-Q water	11.22	-
Total	20	

a The concentration Hemo KlenTaq polymerase enzyme is not provided by the manufacturer.

b There is a lot-to-lot variability in the concentration of GNRs from the manufacturer, but the final concentration is approximately 2.5 nM.

c The DNA comes in a concentrated form, with serial dilutions performed for the specific copy numbers.

**Table 2.** List of primer sets

Primer ID	Sequence (5′–3′)	*T*_m_ (°C)	Amplicon (bp)
CTC–1F	TCCGGAGCGAGTTACGAAGA	57.8	241
CTC–1R	AATCAATGCCCGGGATTGGT	57.4	
CTC–2F	TTGAGAGAACGTGCGGGCGA	61.7	279
CTC–2R	AATGCCCGGGATTGGTTGATCG	60.2	
CTC–3F	GCGGTTGCGTGTCCTGTGACCT	64.4	153
CTC–3R	GCAAATCGCCCGCACGTTCTCT	62.8	
CTC–6F	AGTCCTCTAGTACAAACACCCCCA	59.1	301
CTC–6R	TCGCCCGCACGTTCTCTCAA	61.7	
CTC-WF	GAGATTGAGTGTCCTGCGCGCG	62.8	N/A
CTC-WR	GCAAATCACACACACGGCGCGC	64.5	

In previous publications, we have determined the optimal thermocycling conditions of the plasmonic PCR [15, 16]. We denoted our PCR thermocycling protocols as [denaturation temperature, annealing temperature, elongation temperature] °C and [denaturation time, annealing time, elongation time] seconds, for stage-specific temperatures and holding times, respectively. The thermocycling protocol used in subsequent experiments - denaturation 85°C, 1 s; annealing 58°C, 5 s; elongation 72°C, 1 s – will be referred to as [85–58–72] at a [1–5–1] protocol in this paper. For the most part, only annealing temperatures were varied based on primer design and melting temperature determination (*T*_m_).

### Assessment of the limit of detection (LoD) and limit of blank (LoB)

The LoD was determined following the protocol outlined by Armbruster and Pry [17]. Unless stated otherwise, CTC-6 primers were used in all subsequent assessments including measurements for PCR efficiency, reliability, and replicability (see below). Ten-fold serial dilutions of CTC DNA were prepared ranging from 10^5^ to 10^1^, as well as a no-template control (NTC). Each dilution was amplified 4 times in the POC plasmonic qPCR, and the lowest dilutions returning 4 positive signals were repeated an additional 20 times. The NTC was also repeated 20 times to assess the LoB. All samples were run for 60 cycles, using the 1–5–1 protocol with annealing at 58°C. All reactions were run on 1.5% agarose gels to confirm the fluorescence output signals of DNA amplification of the 4-channel POC plasmonic qPCR.

### PCR efficiency

PCR efficiency was calculated according to the protocol outlined by Evrard et al., [18]. Equations for determining PCR efficiency can be found in Supplemental Data 1.

### Reliability and amplification time replicability

To determine reaction reliability, 24 reactions of 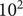 and 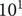 CTC dilutions, respectively, were prepared in a plasmonic PCR mixture and tested consecutively. The average *C*_t_ values for both dilutions and their standard deviations were calculated using an assessment algorithm.

To assess the replicability of amplification time, 32 × 20 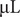 reaction mixtures were prepared, and all 4 channels were run through 30 cycles of amplification 8 times. The times at the final fluorescence collection were collected and averaged.

### Positive from negative sample discrimination

The differentiation of positive from negative template samples is described by true positives (TP) and true negatives (TN), and false positives (FP) and false negatives (FN). A conventional Peltier block-based PCR thermocycler was used to validate the positive CTC cryptic plasmid spiked samples (60 samples in d_2_H_2_O and 20 samples in 2.5% urine, for a total of 80 samples) and negative (26 samples in d_2_H_2_O and 4 samples in 2.5% urine) samples. Once validated, the samples were tested in the Kimera P-IV and compared with the protocol on the conventional platform. Useful equations for discriminating true positive from true negative samples can be found in Supplemental Data 1.

### Urine PCR toxicity and specificity

Dilutions of 20%, 10%, 5%, and 2.5% of stock negative first-void urine (FVU) in d_2_H_2_O were prepared and spiked with *C. trachomatis* DNA to assess the concentrations at which urine exhibited inhibitory effects on PCR amplicon generation and/or fluorescent signal output. All urine samples were provided from healthy volunteers. Experiments using *C. trachomatis* DNA urine were done either using urine diluted to 2.5% of its original concentration (1/40 dilution in d_2_H_2_O) and spiked with DNA, or undiluted urine spiked with CTC DNA and subsequently diluted to 1/40 final concentration. The specificity of the *C. trachomatis* PCR assay was tested using 10^4^ copies of *Neisseria gonorrhoeae* DNA (Quantitative DNA, purchased from ATCC, Manassas, VA, USA) spiked into 1/40 diluted urine.

### Statistical methods and data analysis

Raw data analysis was conducted using a custom Python script, including curve fitting, multi-curve plotting, output curve parameter analysis, and efficiency calculations. *C*_t_ values were determined by fitting sigmoid curves through raw data, normalizing them, and intersecting the linear portion of said curve with the baseline value. Two-sided *t*-tests were conducted on RStudio to analyze trends pertaining to false positive signals generated by primer dimers and discriminate them from true positive signals. All analyzed data showed normalized distributions, equal variance was assumed, and a type I error of *α* = 0.05 was used.

## Results and discussion

### Kimera P-IV 4-channel POC plasmonic qPCR device

**Figure 1.**
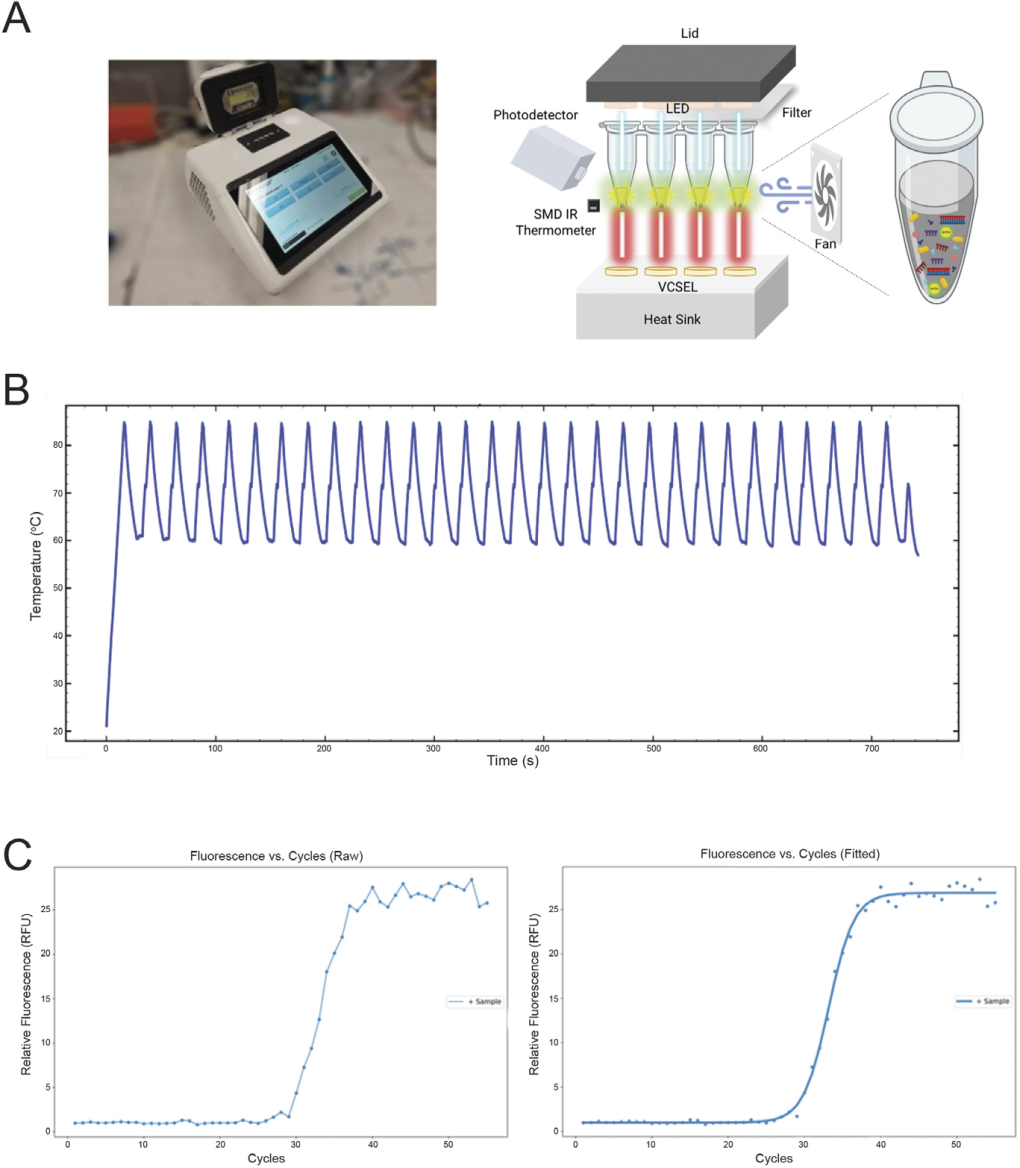
Kimera P-IV Plasmonic PCR Prototype. (a) Image of the 4-channel POC plasmonic qPCR, and the schematic of its functional design. Four 20 μL reaction mixtures in PCR tubes are inserted into the wells. The VCSELs, located directly beneath the reaction mixture, irradiate the GNRs located within the tubes to heat from within. During the cooling phase, the VCSELs turn off, and the fan turns on to cool the samples to the desired annealing temperature. At the end of the annealing phase, the LED in the lid excites the SYTO-16 DNA-binding dye within the mixture, and the photodetector reads the emission signal. Throughout the reaction, the heat sink dissipates unwanted heat generated by the VCSELs. The schematic illustration was created with BioRender.com. (b) Graphical representation of temperature monitoring of 30 PCR cycles conducted by the prototype. Using the 85–60–72°C, 1–5–1 s thermocycling protocol, the reaction is completed in 11.6 min. (c) Real-time quantitative output curves of amplified CTC DNA generated by collected SYTO-16 excitation fluorescent signal. Raw data collected at the end of each annealing cycle and the sigmoid-fitted data are presented.

The prototype was designed and manufactured by Nexless Healthcare (Montreal, QC, Canada). The Kimera P-IV Plasmonic qPCR is encased in a 3D-printed housing, and is lightweight and portable, weighing 1.95 kg. As a qPCR device, genetic material amplification is monitored and displayed in real-time, allowing for immediate test-to-result turnover. An image and graphic representation of the basic functionality of the device are illustrated in Figure 1.

*Heating.* The device uses VCSELs as the source for high-powered 808 nm irradiation as opposed to typical class IV lasers with larger dimensions. As such, space and weight are greatly saved, ultimately contributing to the portability and the POC nature of the device. A singular VCSEL channel can generate 4 W/cm^2^ of power and receives a constant 3.25 A current source. Each VCSEL sits directly below each PCR sample tube and irradiates the GNRs within the sample from the bottom. The VCSELs sit atop a heat sink which dissipates unwanted generated heat, and this sink is located above a hollowed aluminum chamber that has a constant air source blowing through it to further convectively cool the VCSELs (Figure 1a).

*Temperature Monitoring.* The prototype uses non-contact infrared measurements of the sample tube to monitor the bulk sample temperature. To accommodate the compact nature of the device, it uses Melexis’ (MLX90632) 3 × 3 × 1 mm miniature SMD thermometer I, located at a distance of 3 mm in front of each sample tube. The MLX90632 uses a vision window of 1.1 × 1.1 mm with a 50-degree angle field of view, therefore reading from a 2.8 mm × 2.8 mm area of the 20 μL solution. The instrument is calibrated to the bulk temperature of the reaction mix by using a contact fiber optic temperature sensor, and further discrepancies in temperatures are corrected for by melting curve analysis of varying oligonucleotide sizes. Using a single channel, the temperature was constantly monitored and recorded throughout thermocycling, as seen in Figure 1b, where 30 cycles of PCR were conducted in as little as 11.6 min.

*Real-time DNA Amplicon Monitoring.* The DNA-binding dye SYTO-16 is used to monitor amplicon generation in real-time, develop a sigmoid output curve, and ultimately extrapolate a *C*_t_ value. Due to SYTO-16’s excitation spectrum of around 488 nm [19], a blue LED is installed in the lid above the four channels behind a 10 mm × 40 mm short pass anti-reflective coated filter which filters out wavelengths above 487 nm. Similarly, due to the dye’s emission spectrum around 518 nm, a silicon PIN photodiode detector (VEMD8080, Vishay) is located posteriorly to each channel behind a 10 mm × 40 mm long pass anti-reflective coated filter allowing light between 512 nm and 740 nm to pass through. This filter is designed in such a way as to impede incoming 808 nm light from the VCSELs, and blue excitation light. Due to the high-power nature of the VCSELs, some incident light can still enter the filter and register within the photodetector. To address this issue, fluorescent readings are taken while the VCSELs are off at the end of the annealing phase. At each annealing step, five consecutive readings are taken with the excitation LED with those values averaged, recorded, and presented to the user interface. Figure 1c shows the raw generated data and the sigmoidal curve fit through the dataset to allow for instantaneous analysis of the *C*_t_ value, among other parameters.

### Primer design and thermocycling protocol

**Figure 2.**
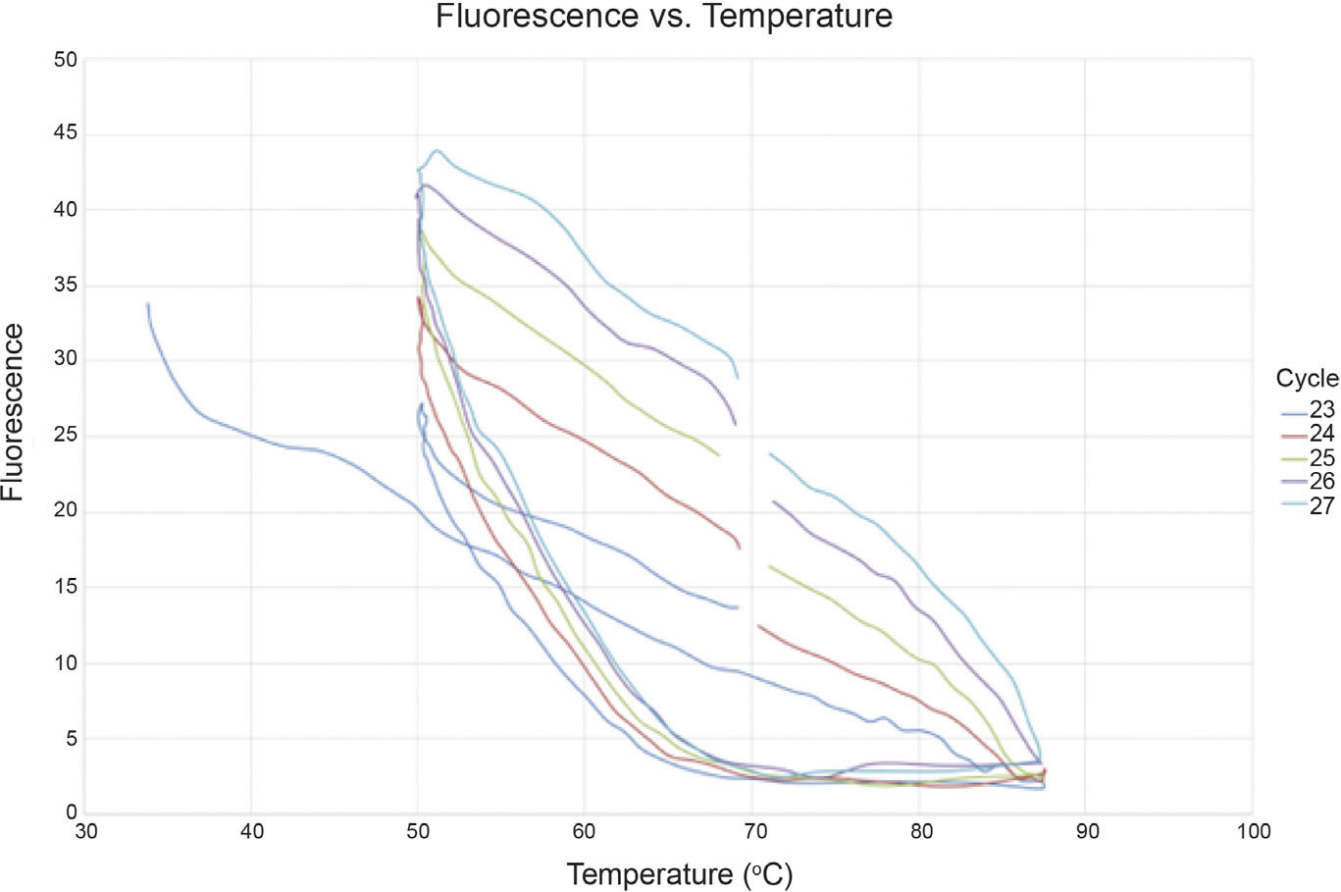
Optimizing thermocycling protocol. Hysteresis curve denoting DNA amplicon strand hybridization and melting represented by measurement of fluorescence from bound and un-bound DNA/SYTO-16 interaction as a function of temperature. Altered to include breaks in the graph to clearly differentiate between cycles. Fluorescence was monitored from 72°C to 88°C (denaturation), 88°C to 50°C (annealing), and 50°C to 72°C (elongation).

The thermocycling temperature protocol used for our device was determined based on output hysteresis curves, which describe DNA de-hybridization and re-hybridization as a function of temperature. Figure 2 describes 5 cycles of PCR beginning at cycle 23 (cycles 23–27) where amplicon generation began producing a discernible fluorescent signal. Fluorescence was monitored at rapid intervals throughout the linear phase of the amplification curve, and the figure reflects SYTO-16’s relationship with double and single-stranded DNA. It is observed that the DNA appears to fully denature around 87°C. De-hybridization increases linearly below 60°C, and annealing temperatures were selected accordingly depending on the primer set’s melting temperature. Therefore, for the *C. trachomatis* amplifications described below, the prototype used an altered thermocycling protocol, with denaturation, annealing, and elongation occurring at 85°C, 58–60°C, and 72°C, respectively. For the subsequently described experiments, the annealing temperature used was 58°C.

### Data analysis and primer design in the context of plasmonic qPCR

**Figure 3.**
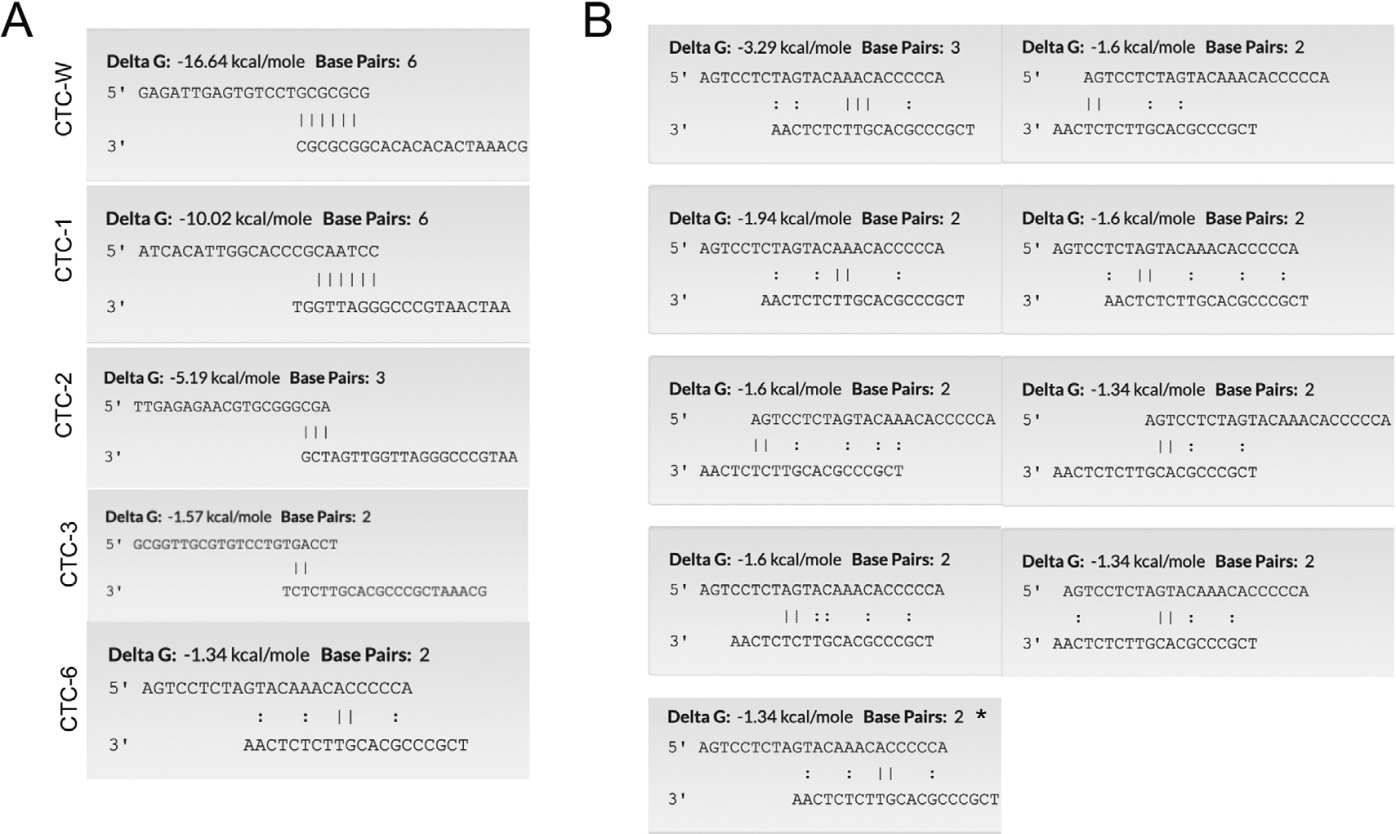
3′ hetero-complementarity binding sequences and the respective Gibb’s free energy changes (Δ*G*) of primers. (a) Highest Δ*G* for all primers that are listed in Table 2. (b) All potential scenarios of CTC6. The highest Δ*G* for CTC6 is marked with an asterisk (*). NCBI Primer-Blast was used for determining primer Δ*G*s, (https://www.ncbi.nlm.nih.gov/tools/primer-blast/index.cgi?GROUP_TARGET=on). Solid lines indicate base-pair matching that contributes to the Δ*G* calculation, while dotted lines do not affect the Δ*G.*

The Kimera P-IV Plasmonic PCR device offers rapid thermocycling compared to traditional platforms, and therefore the biological design considerations are inevitably different. The 5 seconds spent at annealing, compared to the traditional 30 seconds, as well as the rapid rate of heating after annealing, provides an opportunity to avoid primer dimerization [20]. Primers that are more prone to the dimerization effect can also be used at the expense of reduced sensitivity and specificity; however, primer self-extension can be mitigated by selecting primers that have the lowest 3′-hetero complementarity −ΔG value possible. Figure 3 illustrates the highest −Δ*G* determined for each primer pair listed in Table 2. For CTC-6 primers (Figure 3b), all scenarios demonstrate little to no affinity of the primers towards one another at the 3′ ends. Only one situation presents the opportunity for polymerase extension, although primer dimer size would be very small and its effects likely negligible on output fluorescent signal. The free energy change of −1.34 kcal/mol is negligibly low. Therefore, to distinguish a true positive signal from a false positive, especially at low concentrations of the target template, primer design was a crucial consideration. In the context of a rapid plasmonic PCR, the time spent at annealing is shortened and therefore there was an opportunity to mitigate the influence of primer dimerization. Should primer dimers form, distinguishing them from true amplicon is of utmost importance to avoid deeming results falsely positive. Therefore, we analyzed three differentiating parameters, fluorescent ceiling, linear slope of sigmodal curve, and *C*_t_ value, that would distinguish false positives from primer dimerization from true amplicon.

**Figure 4.**
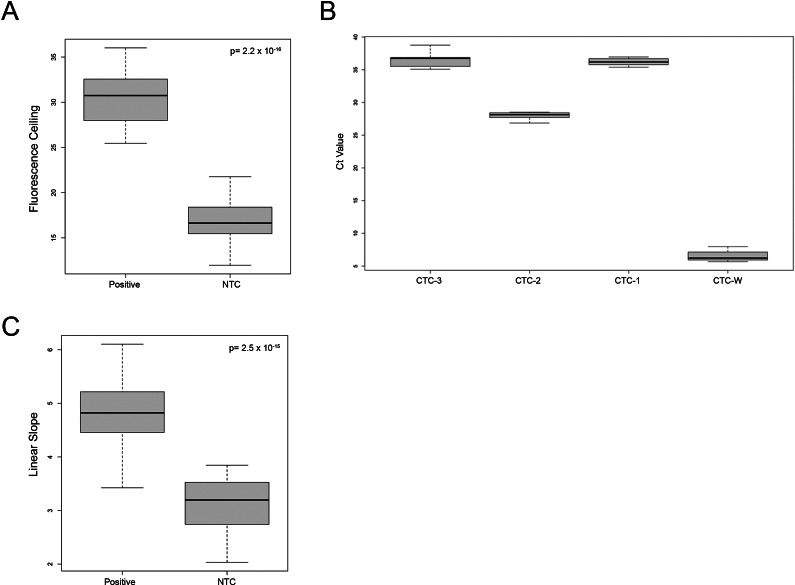
Evaluations to differentiate primer dimer and target amplicon output signals. (a) Boxplots of RFU gain/fluorescence ceiling of positive amplicon and NTC primer dimers using CTC3 primer set. A *P*-value of the difference in means is shown. (b) Boxplots of *C*_t_ values from NTC amplifications were with 4 different primer sets, CTC1, CTC2, CTC3, and CTC-W. (c) Boxplots for the slope of derivative for the linear segment of the sigmoid output curve of positive amplicons and NTC primer dimers of CTC3 primer sets. A *p*-value of the difference in means is shown.

*Fluorescence Ceiling.* The first parameter of differentiation is the relative fluorescence units (RFU) gain of the normalized data. Using CTC3 primer sets in Figure 4a, we observe that the means of the fluorescence ceiling for the positive amplicon and the NTC were 30.37 and 16.81, respectively, with the 95% confidence interval constructed on the RFU of the positive mean is [29.13, 31.6] and [16.16, 17.46] on the NTC mean. The 95% confidence interval of the difference in means is [12.35, 14.77] (*p* = 2.2 × 10^−16^), suggesting that the fluorescence ceiling of the true generated amplicon exceeds that of the primer dimer signal.

The differences in RFU gains reflect the number of fluorescent molecules bound to generated products. Since the size of the CTC3 target amplicon is 153 bp, and that of primer dimers is much less, it is expected that there would be a large difference in the amount of SYTO-16 fluorescent molecules that are bound. It is therefore recommended in plasmonic qPCRs to consider amplicon length ideally above 200 bp when designing primers such that the amplicons can be easily discerned from dimerization.

*Threshold Cycle.* The second parameter of differentiation is the *C*_t_ value. Depending on the primer design, the *C*_t_ value at which the dimer signal develops can vary. Accordingly, we have included NTC controls to assess this value for a particular primer set. The CTC-6F and CTC-6R primers are designed in such a way that the likelihood of hetero- and self-3′ complementarity was reduced as low as possible, denoted by the lowest values of −Δ*G* (see Figure 3a). In Figure 4b, 4 different primer sets (CTC1, CTC-2, CTC-3, and CTC-W) were tested under no template conditions, with their *C*_t_ values at 36.21 ± 0.56, 27.98 ± 0.56, 36.4 ± 1.09, and 6.53 ± 0.78, respectively, all indicating dimerization. It should be noted that CTC-W represents a worst-case scenario set for primer dimer formation with a 6 base-pair hetero-complementarity at the 3′ ends of both forward and reverse primers (i.e., favourable hetero-3′ complementarity/efficient self-extension), resulting in exceptionally low *C*_t_ values. For CTC-6 primers, 27 NTC controls were run in the 4-channel POC plasmonic qPCR for 60 cycles, without the observation of any primer dimers (Supplemental Data 2).

Through the initial cycles of PCR, the sequence complements observed between a primer and target genetic material provide a much larger binding likelihood than that of primer dimers, and it is therefore hypothesized that initial binding efficiencies explain the later *C*_t_ values yielded for primer dimers compared to primer-target binding. Once primer dimer amplicons are produced, the sequence complement would be similar to that of the target sequence, thus, dimerization efficiency should reflect similar outcomes to target amplicon generation efficiency.

*Slope of the Linear Segment of Sigmoid Output Signal.* The last parameter of differentiation is the slope of the linear segment of the output sigmoid curve, located between the growth and saturation segments. Also using CTC3 primer sets, Figure 4c illustrates that the slope of the target amplicon is steeper than that of NTC primer dimers. The mean of the slopes for the positive and NTC samples were 4.82 and 3.14, respectively, with a 95% confidence interval [4.51, 5.13] and [2.90, 3.37], respectively. The 95% confidence interval of the difference in slope means between the 2 groups is [1.375, 1.996] (*p* = 2.5 × 10^−15^), which suggests that the positive samples can be readily differentiated from the NTCs based on the slope of the amplification curve.

The linear portion of the sigmoid output curve demonstrates the rate of amplicon generation from cycle-to-cycle at the most observably quantitative section in the overall reaction. The rationale behind the differences in slopes is hypothesized to be due to differences in fluorophore binding numbers per cycle between target amplicon and primer dimers, and not a function of efficiency discrepancies. Accordingly, during the linear stage of amplification, the longer length of the target amplicon generated per cycle incorporates more fluorophores per cycle compared to primer dimers.

We have previously shown that the Kimera P-IV is capable of melting curve analysis [21]. However, we can now readily show that a false-positive signal generated by a particular primer set can be differentiated from true product amplification, in conjunction with or without the use of melting curve analysis, through the direct analysis of the output signal characteristics: the change in RFU, slope of the linear segment of the sigmoid output curve, and *C*_t_ value. Combining the analysis of these amplification features with melting curve analysis will provide clinicians with further validation of verifying the result outcomes.

### Assessment of Kimera P-IV prototype performance

The LoD and efficiency are important metrics that determine the capabilities of a diagnostics device. They not only give insight into its sensitivity but also into its potential range of applications. A highly sensitive assay will allow for the diagnosis of pathogens with a very low load of genetic material available. In this sense, the efficiency of the device can also be tested. Accordingly, with respect to the Kimera P-IV POC Plasmonic qPCR device, the biological reagent design has been of utmost importance to increase the overall sensitivity to and diagnosis of *C. trachomatis* – the pathogen of choice for the evaluation of this device.

**Figure 5.**
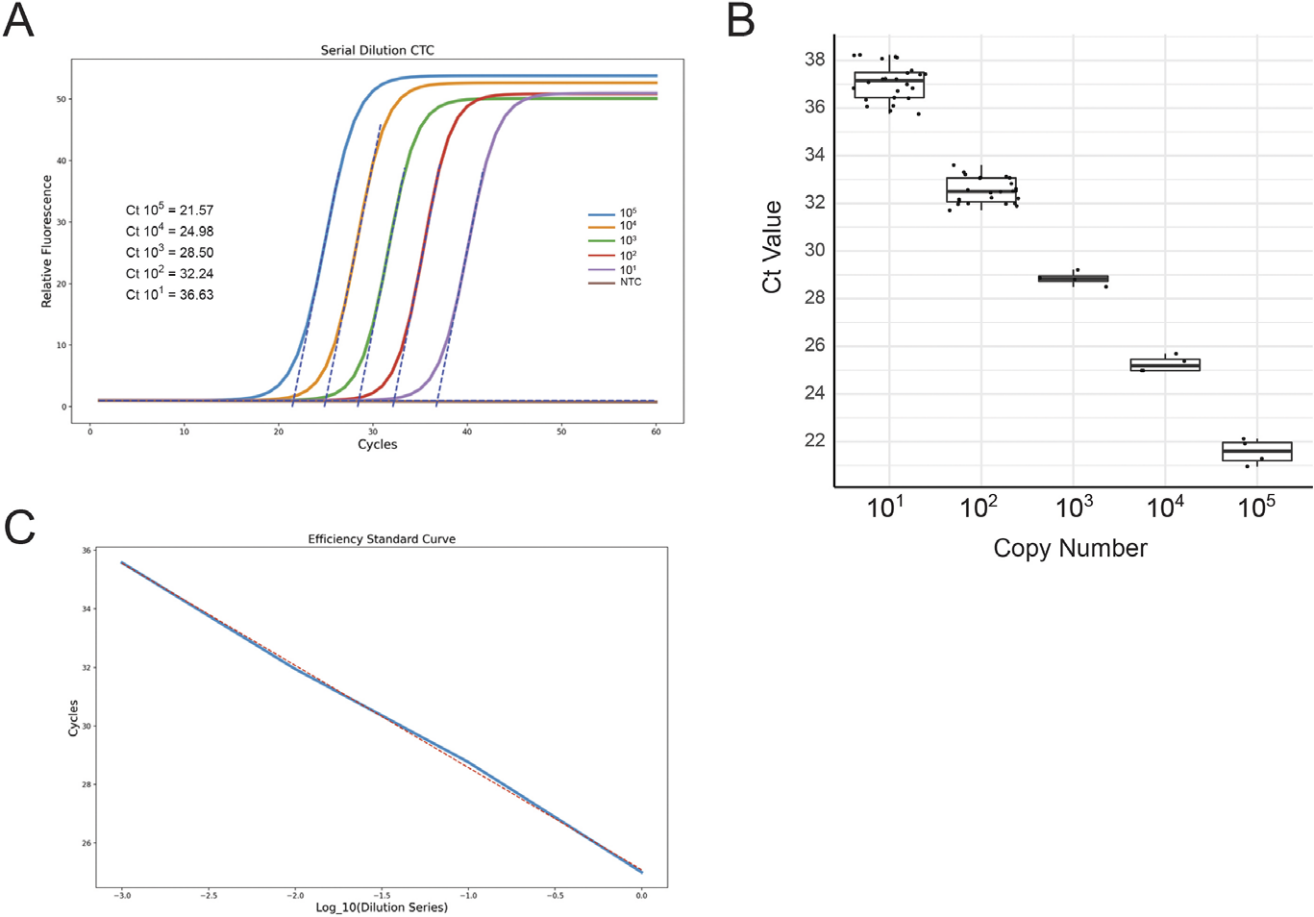
Quantification of *C. trachomatis* DNA. (a) An example of fitted amplification curves of serial dilution of CTC DNA from 10^5^ through 10^1^ copies. Data was fit with a sigmoid curve and *C*_t_ values were extrapolated and presented. *C*_t_ values are defined as the intersection between the relative unit baseline and the slope of the linear segment of the sigmoid output curve. (b) Boxplot of average *C*_t_ values from replicate amplifications of each dilution series. (c) PCR efficiency standard curve of serial dilutions of 10^4^ to 10^1^ CTC DNA copies per reaction, using CTC primers. A trendline is generated through the data.

*Limit of Detection.* Based on the previous experiments regarding the optimization of plasmonic qPCR-specific primer designs, the CTC-6 primer set was selected as they were suitable for a lower target analyte concentration and they seldom generate primer dimers. The results demonstrate a highly sensitive device that can confidently differentiate 10^1^ DNA copies from the NTCs. Figure 5b demonstrates the corresponding average *C*_t_ values of each respective serial dilution from 10^5^ to 10^1^ copies per reaction of target CTC DNA in the PCR mixture, of 21.38 ± 0.54, 25.32 ± 0.35, 28.72 ± 0.3, 32.77 ± 0.53, and 37.03 ± 0.76 (see Supplemental Data 2 for amplification curves). The results from the LoD determination protocol are recorded in Table 3. Positive amplicon generation of each dilution in the series is further validated by gel electrophoresis (see Supplemental Data 3).

**Table 3.** Results of LoD and LoB assessments with the Kimera P-IV

	10^4^ copies	10^3^ copies	10^2^ copies	10^1^ copies	<10^0^ copies	NTC
4× repeats	4/4	4/4	4/4	4/4	4/4	4/4
20× repeats	N/A	N/A	N/A	20/20	20/20	20/20

*PCR Efficiency.* PCR efficiency is a measure of the fraction of target molecules that are copied in any given PCR cycle [18, 22]. While this is generally used to validate qPCR reactions themselves, this is an important assessment of the POC plasmonic qPCR as it provides insight into whether there are unwanted errors made as a result of increased thermocycling speed, or if the device is meeting the research and clinical diagnostics standards. Figure 5c demonstrates a standard curve developed from a single 10-fold serial dilution of CTC plasmid concentration on our platform in a standard plasmonic PCR reaction mix. A linear trendline is fitted to the data, and the slope is used to determine the efficiency. Twelve dilution series were conducted from 10^4^ to 10^1^, and the average efficiency value was found to be 88% with a standard deviation of 6.71%.

These results demonstrate that the Kimera P-IV prototype generates 88% of the theoretical expected amplicon per amplification cycle, and is competitive within the 90–110% of other rapid devices [23]. We postulate that any losses in efficiency are at the level of rapid heating/cooling rates and reduced hold times. It is also important to consider biological reagent optimizations, as efficiency can be affected by amplicon length, primer hybridization efficiencies, and polymerase enzyme speed; however, a standard deviation of 6.71% indicates the consistency in the machine’s ability to generate similar efficiency values.

*Reliability.* For a diagnostic device to be considered a valid tool to assess a construct, it must also be reliable. Reliability is a measure of reproducibility, an important variable to assess when validating a new diagnostic device. This ensures that the device yields replicable results with high confidence between each run. To evaluate the reliability of the Kimera P-IV Prototype device, 24 reactions of 10^2^ and 10^1^ CTC dilutions were prepared and tested on the device. The 10^2^ dilutions yielded an average *C*_t_ value of 32.53 ± 0.53, and the 10^1^ dilutions yielded an average *C*_t_ value of 37.07 ± 0.76. The low standard deviation values indicate very high replicability, with results falling within less than 1 *C*_t_ value between runs.

*Amplification Time Replicability.* Another important characteristic of a POC device is its ability to complete thermocycling in a consistent and reliable time span. This assessment reflects the machine’s robustness, consistency, and hardware and software reliability. To evaluate the amplification time replicability of the Kimera P-IV Prototype device, using CTC6 primer sets, 6 samples from the same mixture were run consecutively for 30 cycles, and the time to completion was measured. For the 6 runs tested, an average of 13.98 min was required to complete 30 cycles with a standard deviation of 0.38 min, which indicates consistent assay time replicability from run-to-run. All 4 channels were run simultaneously during each of the 6 runs, and as they are all regulated by independent VCSELs, a synchronization period was implemented at 2°C below denaturation to ensure similar finishing times between each channel and maximized fan-induced cooling time (since a single fan cools all channels simultaneously). Table 4 highlights the results of the individual trials used to assess the amplification time replicability.

**Table 4.** Reliability of amplification time

Run	1	2	3	4	5	6	Average	SD
Time (min)	13.29	13.97	14.38	14.30	13.93	13.98	13.98	0.38

*Positive versus Negative Sample Discrimination.* Discerning positive from negative samples is an important benchmark test when validating a novel diagnostic device to ensure that positive and negative samples can be properly diagnosed, which is essential for a successful translation into clinical settings. To verify the Kimera P-IV Prototype device’s discernability in diagnosing *C. trachomatis*, we have used a conventional Peltier block-based thermocycler to perform PCR reactions and compared the results with the plasmonic qPCR device (Table 5). A sensitive and specific device will properly confirm the results of the gold-standard test with high accuracy.

**Table 5.** Results of positive from negative sample discrimination, and specificity assessments with the Kimera P-IV

	Positive reference test	Negative teference test	Total
Plasmonic positive	TP = 80	FP = 0	80
Plasmonic negative	FN = 0	TN = 30	30
Total reactions	80	30	110

Summary of the positive from negative sample discrimination and specificity assessments for 110 total samples. Reference test refers to positive and negative samples verified by with conventional PCR machine. The 80 positive samples are divided as follows (in a final reaction volume of 20 μL): 8 samples of 10^5^ copies, 8 samples of 10^4^ copies, 8 samples of 10^3^ copies, 28 samples of 10^2^ copies, 28 samples of 10^1^ copies.

TP = true positive, FP = false positive, FN = false negative, TN = true negative

As indicated, the device discriminated 100% of positive template samples from negative and did not yield false positive or false negative results. This level is attainable in conjunction with the nature of the primer design, allowing for the discrimination between a <10^1^ sample and an NTC by ensuring a low affinity for dimerization. The positive or negative natures of the samples at varying concentrations of CTC DNA were primarily determined via conventional PCR and subsequently confirmed on our platform.

### Spiked urine sample analysis

In a clinical scenario, patient samples often contain various inhibitory molecules that can affect PCR [24]. While different sample preparation methods have been developed to improve the purity and yield of the nucleic acid from the samples, these processes take time and often require a centralized laboratory. On the other hand, increasing numbers of publications have been focusing on an extraction-free, direct PCR approach in which samples are placed directly under a thermocycler without a clean-up step [25, 26]. Moreover, it has also been shown that direct PCR analysis of *C. trachomatis* could be performed from endocervical swabs by initially boiling the samples [27, 28]. We have also been able to demonstrate direct and extraction-free plasmonic PCR amplification from a bacterial culture [16].

**Figure 6.**
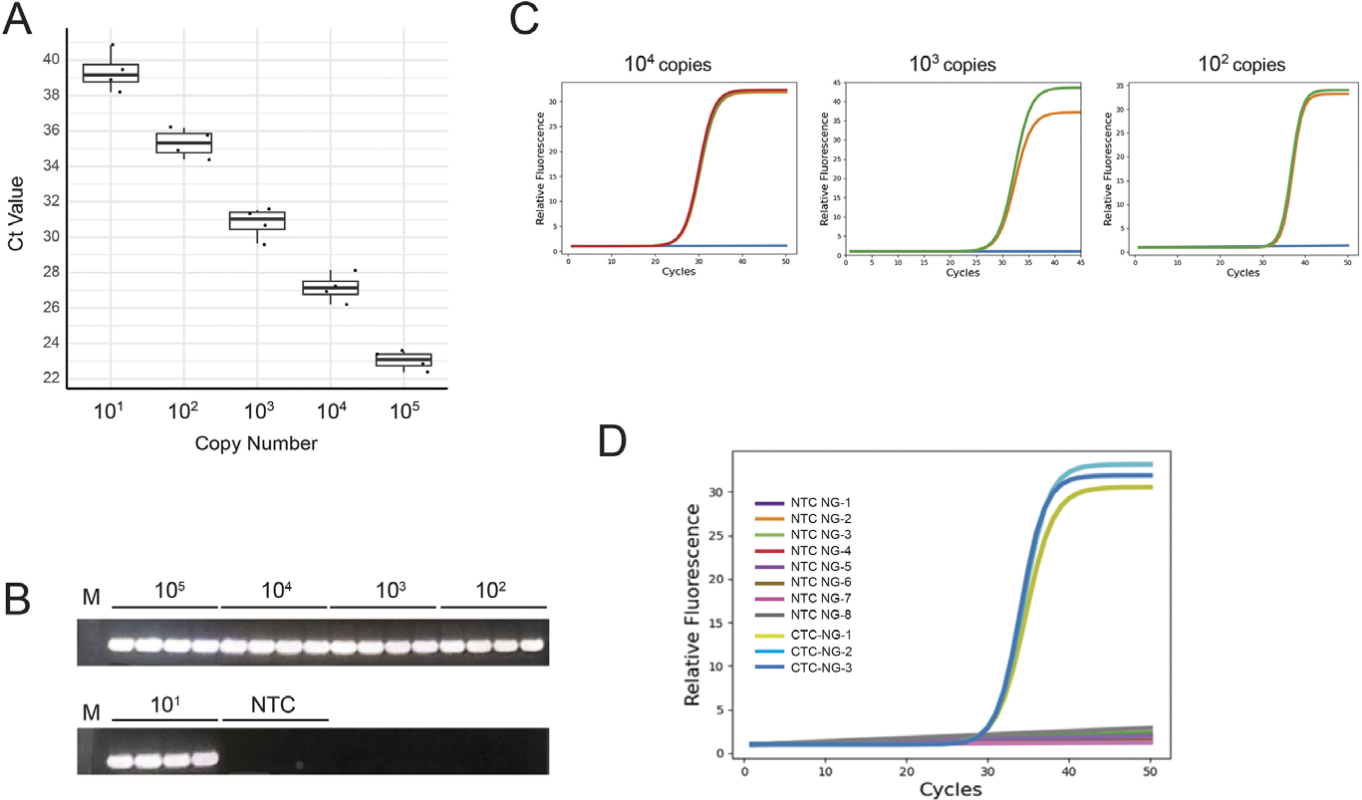
Spiked urine samples. (a) Boxplots of average *C*_t_ values of 1/40 diluted urine samples spiked with CTC DNA dilutions, ranging from 10^5^ to 10^1^ copies. Each graph represents 4 repeats of each dilution. (b) Corresponding gel electrophoresis results. (c) Amplification curves of undiluted urine spiked with 10^4^, 10^3,^ or 10^2^ copies of CTC DNA, subsequently diluted to 1/40 (*n* = 4 each). (d) *C. trachomatis-specific* amplification reactions were carried out with diluted urine spiked with 10^4^ copies of *N. gonorrhea* (NTC NG, *n* = 8), Illustrated on the amplification curves are also positive reactions of diluted urine spiked with 10^2^ copies of *C. trachomatis* and 10^4^ copies of *N. gonorrhea* (CTC-NG, *n* = 3).

Urine samples are often collected for *C. trachomatis* testing, therefore the ability to amplify spiked genetic material in patient urine directly in the machine is a crucial feature of our POC device. This will drastically reduce sample preparation time and allow for a rapid test-to-result turnover time. We first determined the dilution limit and found that a 1/40 dilution did not inhibit plasmonic PCR (see Supplemental Data 4). These experiments were reproduced with different DNA template numbers, urine dilutions, urine samples that ranged in color and turbidity, and positive water controls, wherein all continually supported PCR amplification from all sample types (data not shown). Therefore, to demonstrate the compatibility of our system under a “mock” clinical scenario, we tested 20 urine samples from individual patients spiked with CTC DNA (4 samples of each dilution spanning 10^5^ to 10^1^ copies/reaction), and 4 NTCs on the Kimera P-IV prototype (Figure 6, see Supplemental Data 5 for amplification curves). The average *C*_t_ values for urine samples were 23.03 ± 0.52, 27.15 ± 0.80, 30.82 ± 0.86, 32.54 ± 0.81, and 39.35 ± 1.11 for each dilution indicating that there may be minimal losses in *C*_t_ value when comparing urine to water samples. The *C*_t_ value discrepancies between spiked urine and LOD water samples for each dilution are 1.65, 1.83, 2.10, 0.23, and 2.30, respectively. These differences can be attributed to mild inhibitory effects of the contents within the urine, where the slight variability is a consequence of direct urine versus purified nucleic acid analysis. Additionally, the positive amplicon of spiked urine samples demonstrated strong fluorescent output signals, while the NTCs did not. Differences in the average RFU between urine-spiked and water samples were not significant, and therefore, the urine does not affect the intensity of the fluorescent signal at the specified dilutions. Additionally, we also directly spiked undiluted urine with 10^4^, 10^3,^ and 10^2^ copies of CTC DNA and carried out subsequent final dilutions of 1/40, with the final copy number representing 3000, 300, and 30 copies/reaction, respectively. Similarly, we continued to observe robust amplification with comparable *C*_t_ values (Figure 6c, see Supplemental Data 5).

Testing of *C. trachomatis* is normally performed in conjunction with *N. gonorrhoeae.* To assess the parameters of the specificity of our CTC amplification assay, 10^4^ copies of *N. gonorrhoeae* DNA were spiked into 1/40 diluted urine (NTC NG) and were compared to diluted urine samples containing 10^2^ copies of CTC and 10^4^ copies of *N. gonorrhoeae.* Amplification was only observed in the 10^2^ copies of CTC-spiked sample controls, and not in NTC NG controls (Figure 6d). Although this is a limited pathogen specificity test of our assay, urine also contains shed epithelial cells and fragments of human genomic DNA that may also serve as potential contaminants for our assay; however, we do not observe any aberrant amplification to our *C. trachomatis* assay, with all urine only controls always giving null results. In lieu of extensive access to genetic material from other pathogens, we performed a bioinformatic analysis of our CTC-6 designed primers, with the results indicating that they only show sequence specificity for *C. trachomatis* strain and no other pathogen. This type of *in silico* application is being readily applied to design PCR assays for unique pathogen identification or to differentiate strains within the same family [29–33].

This assessment gives excellent insight into the capabilities of the machine and its potential translation into clinical settings; supported by our preliminary investigations of using diluted urine for PCR analysis for *C. trachomatis* [34]. Future evaluations will require clinical specificity and sensitivity studies, including true positive clinical urine, and vaginal and anal swab samples using our Kimera P-IV platform.

## Conclusions

By far the greatest infectious disease concern is the emergence of antibiotic resistance in multiple microbial pathogens, both total resistance and resistance towards common antibiotics. Health Canada and the Centers for Disease Control and Prevention (US) point out the grave repercussions of antibiotic resistance in the United States (2 million infections annually) and define several organisms as urgent threats [35, 36]. WHO has also issued a recent (June 2020, http://www.who.int/mediacentre/factsheets/antibiotic-resistance/en/) priority list of 20 bacterial pathogens displaying worrisome antimicrobial resistance [37–40]. Included in this group is the extremely perturbing resistance to the third-generation cephalosporins documented in *Neisseria Gonorrhoeae* [10, 41].

The vast majority of emerging antibiotic resistance results from the long practice of employing broad-spectrum antibiotics against pathogens [42]. Implementation of broad-spectrum antibiotics is standard practice where specific pathogens are not yet characterized or when clinical judgment suggests a possible bacterial pathogen (at least 50% of antibiotics prescribed are not needed or misused) [43]. Such widespread use has had an enormous selection pressure on nearly all microbial to develop resistance against currently available antibiotics. This has been compounded by the lack of new antibiotic drug development, as effective targets against the pathogens are becoming more difficult to define. Therefore, to address these practices, antibiotic stewardship programs are being urgently instituted worldwide to encourage responsible antibiotic practices [44–48].

*C. trachomatis* is the most commonly diagnosed infectious disease in Canada with increasing rates in the last decade and has reached over 372.9 cases per 100,000 of the Canadian population in 2020 [12]. Given that these are overwhelmingly STDs, individuals mostly present themselves to infectious disease clinics. This clinical setting would be the ideal location to conduct plasmonic PCR testing. PCR has become the standard method used to diagnose STD infections, and we have shown that our device has the potential to perform direct sample analysis via a small urine dilution. Therefore, a plasmonic PCR diagnostic POC device such as the Kimera P-IV would have a dramatic effect, allowing for near-instant pathogen detection and subsequent appropriate dispensing of antimicrobial agents at the site where the patient is directly and initially evaluated by the physician. Not only would this allow immediate and appropriate antibiotic usage on the spot, but it would also have a beneficial effect on reducing antibiotic resistance.

The Kimera-P-IV device can conduct ultra-rapid PCR with real-time result generation in a direct patient setting. The features that we have described are all made possible through the irradiation of gold nanorods by VCSEL technology, and cycle-to-cycle monitoring of DNA-binding dye excitation. This device introduces an aspect of the accuracy of detection that is often sacrificed at the expense of speed, achieving an LoD of <10^1^, and a PCR efficiency of 88%, while demonstrating an ability to discern true positive from true negative samples with 100% accuracy, and conduct direct-urine analysis. Further clinical validations are necessary; however, the results presented here within are tremendously promising, and the implementation of this device into clinical settings can tackle glaring issues in the healthcare system, from pandemic control to antimicrobial resistance mitigation.

## Supporting information

Hayes et al. supplementary material Hayes et al. supplementary material

## Data Availability

All data generated and analyzed during this study are available from the corresponding authors upon reasonable request.

## Supplementary material

For supplementary material accompanying this paper visit http://doi.org/10.1017/S0950268825000081. click here to view supplementary material
