# Supplementary material for: Performance and functional assessment of the Kimera P-IV point-of-care plasmonic qPCR prototype for ultra rapid pathogen detection of chlamydia trachomatis
Source: Epidemiol Infect. 2025 Jan 30;153:e27. doi: 10.1017/S0950268825000081 (PMC11869076; doi:10.1017/S0950268825000081)
Supplement: Hayes et al. supplementary material [file S0950268825000081sup001.pdf]

Performance and Functional Assessment of the Kimera P-IV Point-of-Care Plasmonic qPCR Prototype for Ultra Rapid Pathogen Detection of *Chlamydia Trachomatis*

Joshua Hayes [1,2], Seung Soo Lee [1,2], Jason Carnevale [3], Daniel Shamir [4], Marc Bohbot [4], Andrew G. Kirk [5];

Miltiadis Paliouras [2,6]§, Mark Trifiro [1,2,6]§

1. Lady Davis Institute for Medical for Medical Research – Jewish General Hospital, Montreal, QC, Canada
2. Division of Experimental Medicine, McGill University, Montreal, QC, Canada
3. Department of Biology, Concordia University, Montreal, QC
4. Nexless Healthcare LP., Montreal, QC, Canada
5. Department of Electrical and Computer Engineering, McGill University, Montreal, QC, Canada
6. Department of Medicine, McGill University, Montreal, QC, Canada

§ Corresponding authors

Miltiadis Paliouras, Ph.D.
McGill University
Department of Medicine
Division of Experimental Medicine
Montreal, QC, Canada

miltiadis.paliouras@mcgill.ca

Mark Trifiro, M.D.
McGill University
https://apps.ladydavis.ca/RCMS/Department of Medicine
Lady Davis Institute for Medical Research – Jewish General Hospital
Montreal, QC, Canada
mark.trifiro@mcgill.ca

Supplemental Data 1. Expanded Materials and Methods.

PCR Efficiency

From by Evrard et al., [1]. Amplification is theoretically calculated by:

$$\log Q = \ log\ q_o + n \log(1+\rho)$$

where 'Q' is the final amplicon generated at some amplification cycle n, q_o is the known starting DNA copy number, and ρ is the efficiency coefficient.

Plotting this relationship using serial dilutions of relative fluorescence units (RFU) as a function of Ct value, an arbitrary value of RFU intersecting the linear portions of serial dilution sigmoid output curves was selected and assigned a unit value, and the corresponding cycle values were recorded. These cycle values were then plotted against the $\log_{10}$ (initial DNA concentration), and a standard curve was developed according to the relationship:

$$n = \frac{\log Q}{\log\,(1+\rho)} - \frac{\log q_o}{\log\,(1+\rho)}$$

Sensitivity and Specificity

Sensitivity and specificity are calculated as:

$$sensitivity = \frac{TP}{TP+FN}\,,\ specificity = \frac{TN}{TN+FP}$$

TP = true positives

TN = true negatives

FP = false positives

FN = false negatives

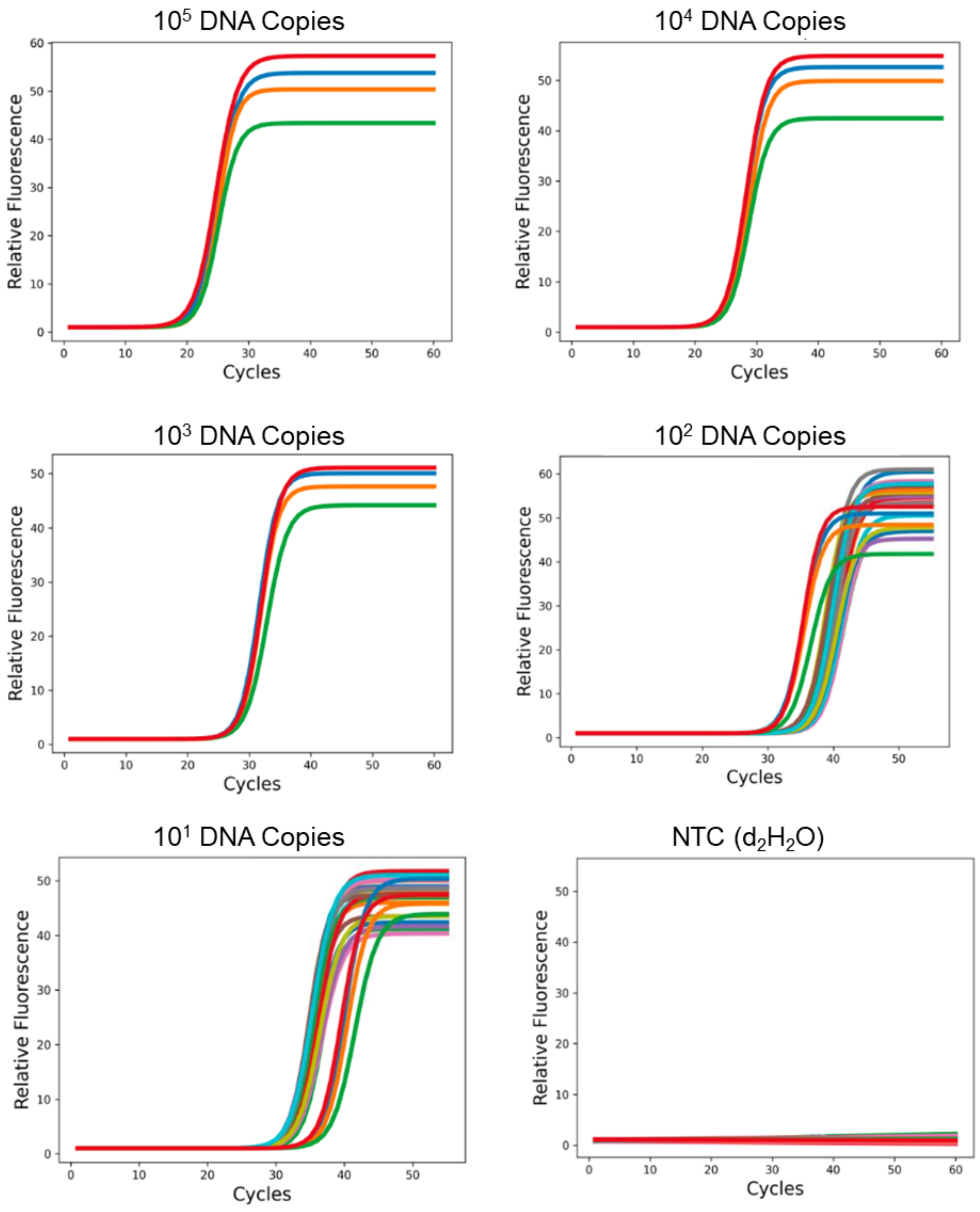

Supplemental Data 2. *C. trachomatis* CTC DNA amplification curves. Illustrated are the amplification curves of serial dilutions of *C. trachomatis* from Figure 5. Replicate of fitted data from each dilution series, of serial dilution of CTC DNA from 10^5 through 10^1 copies and no template controls (NTC).

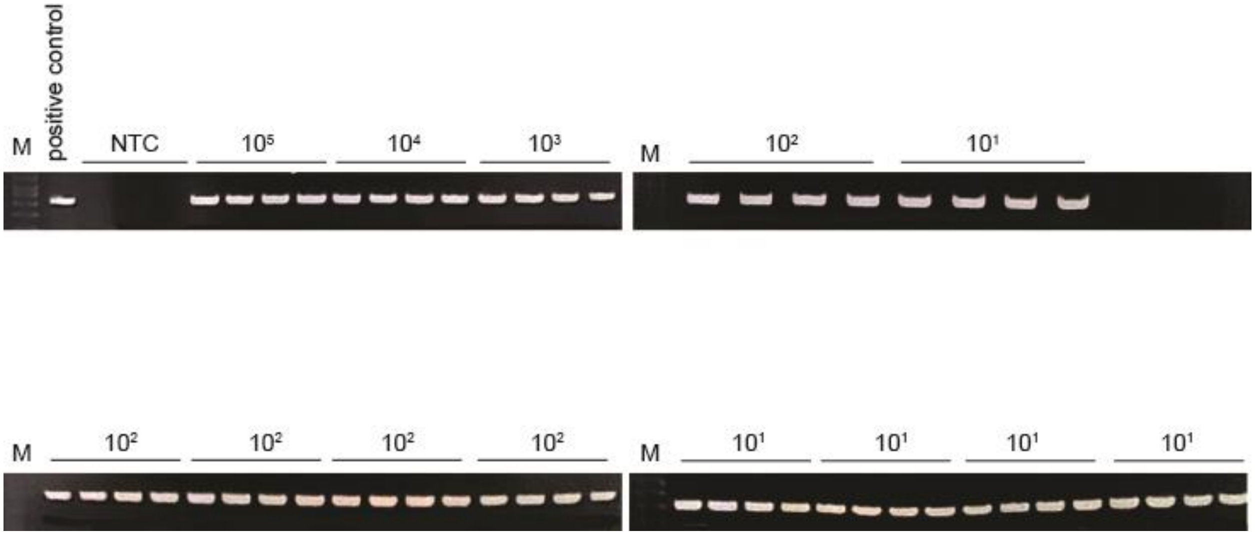

Supplemental Data 3. *C. trachomatis* gel electrophoresis blots. Illustrated are the gel electrophoresis blots of serial dilutions of *C. trachomatis*, from Figure 5.

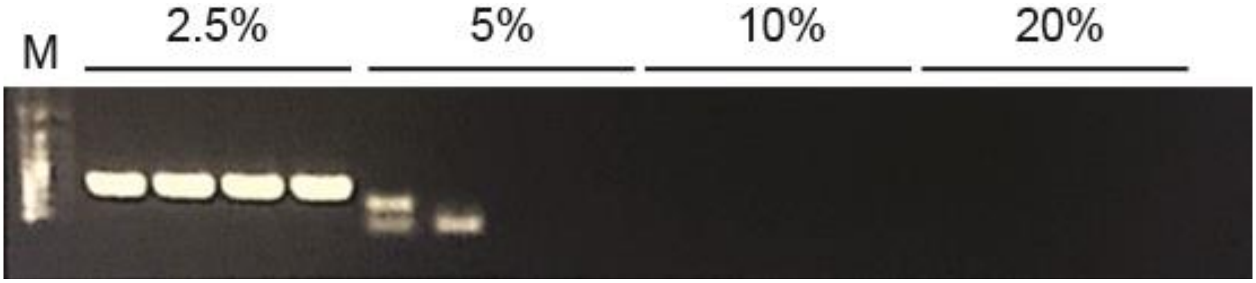

Supplemental Data 4. Urine inhibition. C. trachomatis spiked urine samples to identify the urine dilution that no longer inhibited plasmonic PCR. Urine dilutions were performed in water.

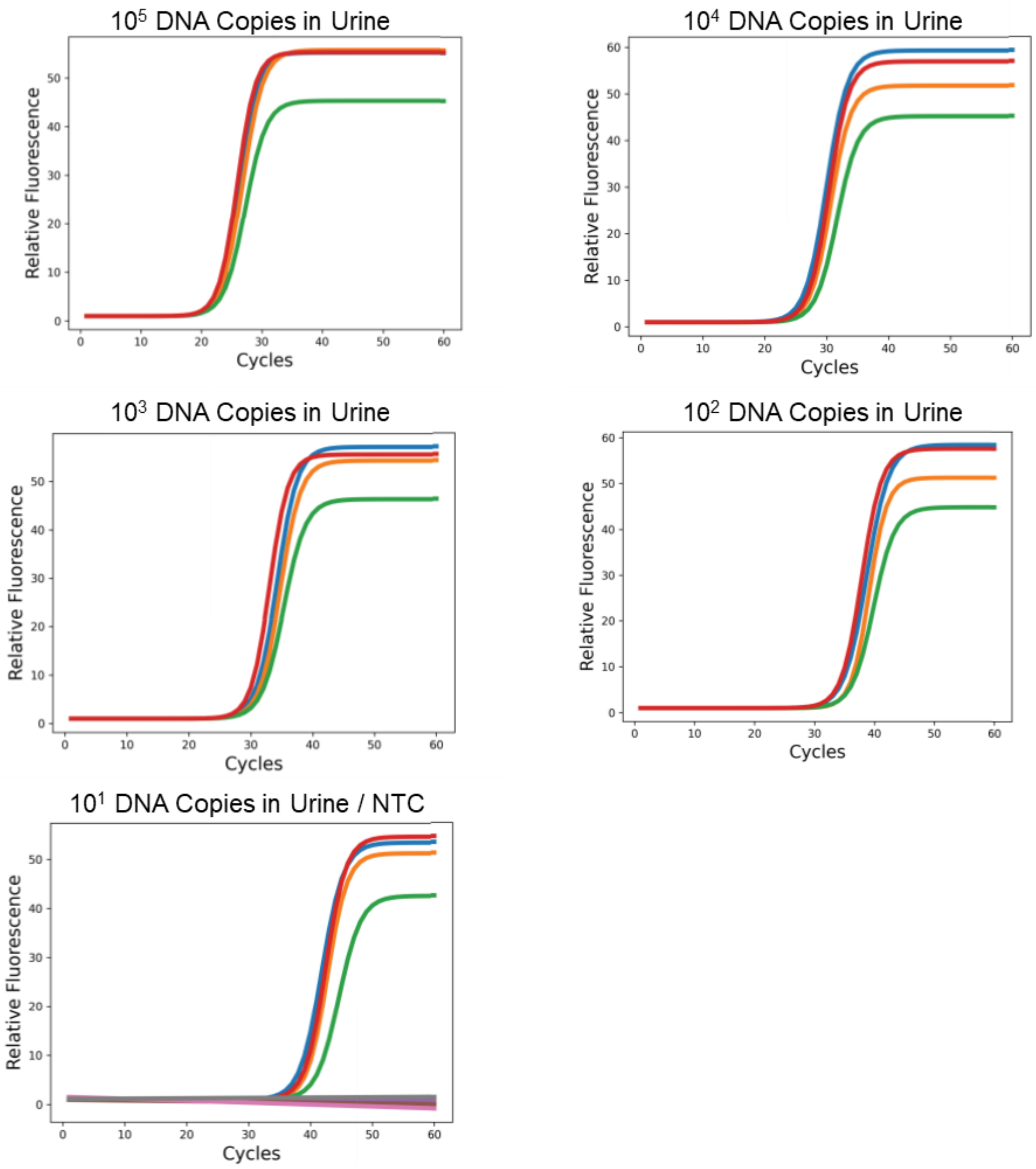

Supplemental Data 5. Spike urine *C. trachomatis* CTC DNA amplification curves. Illustrated are the amplification curves of dilution series of spiked *C. trachomatis* urine samples from Figure 6. Amplification curves of clinical urine samples spiked with CTC DNA dilutions, ranging from 10^5 to 10^1 copies, including 4 NTC samples. Each graph represents 4 repeats of each dilution, and the NTC fluorescence curves were combined the 10^1 dilutions.

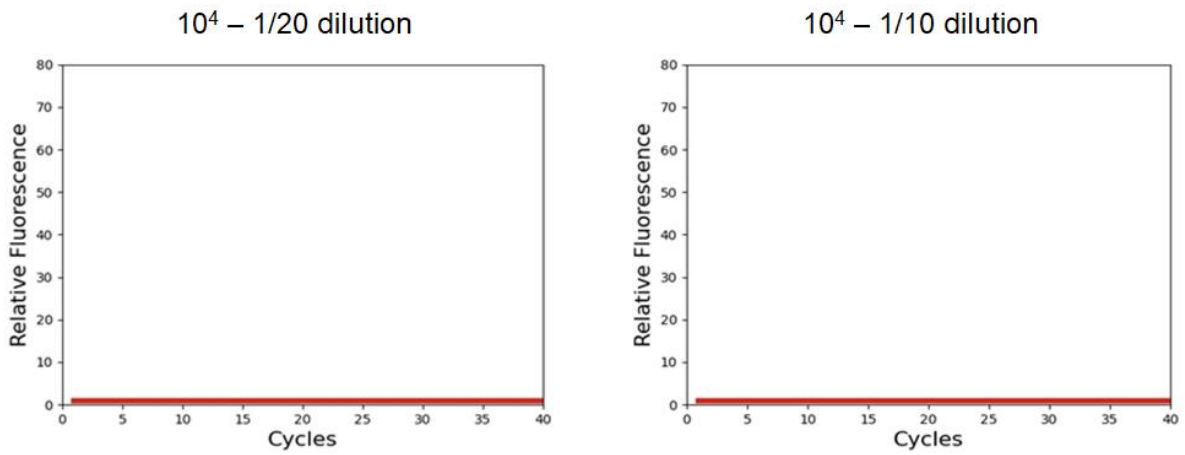

Supplemental Data 5B. Amplification curves of *C. trachomatis* DNA spiked into undiluted urine. Curves represent 10^4 copies of CTC DNA spiked directly into urine and proceeded with carrying out 1/10 and 1/20 final dilution prior to PCR reaction. Results demonstrate either direct or post-dilution of DNA into urine, a minimum final diluted urine concentration of 1/40 is needed for a positive reaction (see Figure 6).

REFERENCES

1. Evrard, A., N. Boulle, and G.s. Lutfalla, *Real-Time PCR*. Nanoscience: Nanobiotechnology and Nanobiology, ed. P. Boisseau and M. Lahmani. 2009: Springer Science & Business Media.
